# Can the choice reaction time be modified after COVID-19 diagnosis? A prospective cohort study

**DOI:** 10.1590/1980-5764-DN-2021-0116

**Published:** 2022-05-23

**Authors:** Gustavo José Luvizutto, Angélica Taciana Sisconetto, Pablo Andrei Appelt, Kelly Savana Minaré Baldo Sucupira, Eduardo de Moura, Luciane Aparecida Pascucci Sande de Souza

**Affiliations:** 1 Universidade Federal do Triângulo Mineiro, Departamento de Fisioterapia Aplicada, Uberaba, MG, Brazil.; 2 Universidade Federal do Triângulo Mineiro, Departamento de Ciência do Esporte, Uberaba, MG, Brazil.

**Keywords:** COVID-19, Reaction Time, Cognition, COVID-19, Tempo de Reação, Cognição

## Abstract

Assessment of cognitive processing speed through choice reaction time (CRT) can be an objective tool to assess cognitive functions after COVID-19 infection.

**Objective:**

This study aimed to assess CRT in individuals after acute COVID-19 infection over 1 year.

**Methods:**

We prospectively analyzed 30 individuals (male: 9, female: 21) with mild-moderate functional status after COVID-19 and 30 individuals (male: 8, female: 22) without COVID-19. Cognitive and neuropsychiatric symptoms were evaluated using the Montreal Cognitive Assessment (MoCA) and Hospital Anxiety and Depression Scale (HADS), respectively. CRT (milliseconds) was evaluated by finding the difference between the photodiode signal and the electromyographic (EMG) onset latency of anterior deltoid, brachial biceps, and triceps during the task of reaching a luminous target. CRT was evaluated three times over 1 year after COVID-19: baseline assessment (>4 weeks of COVID-19 diagnosis), between 3 and 6 months, and between 6 and 12 months.

**Results:**

The multiple comparison analysis shows CRT reduction of the anterior deltoid in the COVID-19 group at 3-6 (p=0.001) and 6-12 months (p<0.001) compared to the control group. We also observed CRT reduction of the triceps at 6-12 months (p=0.002) and brachial biceps at 0-3 (p<0.001), 3-6 (p<0.001), and 6-12 months (p<0.001) in the COVID-19 compared to the control group. Moderate correlations were observed between MoCA and CRT of the anterior deltoid (r=-0.63; p=0.002) and brachial biceps (r=-0.67; p=0.001) at 6–12 months in the COVID-19 group.

**Conclusions:**

There was a reduction in CRT after acute COVID-19 over 1 year. A negative correlation was also observed between MoCA and CRT only from 6 to 12 months after COVID-19 infection.

## RESUMO

A avaliação da velocidade de processamento cognitivo por meio do tempo de reação de escolha (TRE) pode ser uma ferramenta objetiva para acompanhar as alterações cognitivas após a COVID-19.

**Objetivo:**

Avaliar o TRE em pacientes após infecção aguda por COVID-19 ao longo de um ano.

**Métodos:**

Foram avaliados 30 indivíduos (sexo masculino: nove; feminino: 21) com estado funcional leve-moderado após infecção por COVID-19 e 30 (sexo masculino: oito; feminino: 22) sem COVID-19. A avaliação foi feita pelo *Montreal Cognitive Assessment* (MoCA) e pela Escala Hospitalar de Ansiedade e Depressão. O TRE (milissegundos) foi avaliado pela diferença entre o sinal luminoso e a latência de início da atividade muscular (EMG) do deltoide anterior (DA), do bíceps braquial (BB) e do tríceps durante uma tarefa de alcance. O TRE foi avaliado ao longo de um ano: avaliação inicial (>4 semanas após diagnóstico de COVID-19), em 3–6 meses e em 6–12 meses.

**Resultados:**

Houve redução do TRE do DA no grupo COVID-19 em 3–6 meses (p=0,001) e 6–12 meses (p<0,001) em comparação com o grupo de controle. Também foi observada redução na TRE do tríceps em 6–12 meses (p=0,002) e do BB em 0–3 meses (p<0,001), 3–6 meses (p<0,001) e 6–12 meses (p<0,001) no grupo COVID-19 em comparação com o grupo de controle. Correlações moderadas foram observadas entre MoCA e TRE do DA (r=-0,63; p=0,002) e BB (r=-0,67; p=0,001) aos 6–12 meses no grupo COVID-19.

**Conclusões:**

Houve redução do TRE após COVID-19 ao longo de um ano, além de correlação negativa entre MoCA e TRE no período de seis a 12 meses após COVID-19.

## INTRODUCTION

Some studies have demonstrated associations between severe acute respiratory syndrome coronavirus 2 (SARS-CoV-2) infection and neurological dysfunction in the early phase^1^ and long term, mainly cognitive deficits in executive function, attention, language, and delayed recall^2,3^. The virus can enter the cerebral circulation by interacting with the angiotensin-converting enzyme-2 (ACE-2) receptor and infect neural cells^3,4^ or cross the blood-brain barrier and activate the brain’s immune cell to produce neural cytokines, leading to brain dysfunction^5^.

The spread and persistence of the virus in brain cells remains debatable. However, several studies have observed that coronavirus disease 2019 (COVID-19) can change brain activity and connectivity^6–8^, causing cognitive dysfunction for months after infection^9,10^. In addition, there are increasingly frequent reports of memory impairment, concentration difficulties, and long-term neuropsychiatric symptoms^11,12^. This long COVID-19 status is defined as “brain fog,” which is the cognitive complaint of slow and confused thinking^9,10^.

Assessment of cognitive processing speed through choice reaction time (CRT) can be an objective tool to assess brain fog after acute COVID-19. Decision-making is the reaction time (RT) for more than one visual stimulus (choice RT) and the onset of muscle activity to assess cognitive function and processing speed^13^. The CRT process includes many cognitive functions, such as recognition, association, coordination, inhibition, and decision planning stages^14,15^. These cognitive changes after the acute period of COVID-19 can have long-term negative impacts, resulting in cognitive, behavioral, and emotional changes^9,10^.

Long-term monitoring of neurological and cognitive function in individuals after COVID-19 infection is necessary to understand changes in cognitive behavior and verify possible neurodegenerative diseases. In addition, Hellmuth et al. showed that cognitive deficits were not captured by common cognitive screens, such as the Mini-Mental State Examination or Montreal Cognitive Assessment (MoCA), suggesting that systematic and objective cognitive tests can be more beneficial after acute COVID-19^16^. Therefore, this study aimed to assess CRT in individuals with acute COVID-19 after over 1 year. In addition, the correlation between MoCA, anxiety, depression, and CRT was also evaluated in the COVID-19 group.

## METHODS

### Study design, setting, and participants

This was a 12-month prospective cohort study of individuals with acute COVID-19 in Uberaba, Minas Gerais, Brazil. The research was conducted at the Laboratory of Neuroscience and Motor Control of the Universidade Federal do Triângulo Mineiro (UFTM) between September 2020 and July 2021.

We prospectively analyzed 60 individuals (30 individuals with SARS-CoV-2 laboratory-positive [SARS-CoV-2+] and 30 individuals with SARS-CoV-2 laboratory-negative [SARS-CoV-2-]) who met the study inclusion criteria. The diagnosis of COVID-19 was confirmed by SARS-CoV-2 reverse transcription-polymerase chain reaction of nasopharyngeal swabs and/or SARS-CoV-2 antibody testing. Among the 60 participants, 30 participants had a positive result for SARS-CoV-2 infection (SARS-CoV-2+), while 30 participants had a negative result for SARS-CoV-2 infection (SARS-CoV-2-).

Individuals diagnosed with COVID-19 were recruited from the Uberaba Municipal Health Department and the Clinical Hospital of the Universidade Federal do Triângulo Mineiro. The control group was recruited via radio, television, and digital media. The control group criteria are that they should be negative for COVID-19 at the time of evaluation and should not have a positive diagnosis of COVID-19 since the beginning of the pandemic. This study was approved by our institutional review board (CAAE: 30684820.4.0000.5154).

### Eligibility criteria

We included individuals with mild to moderate functional status after COVID-19 (grades 0–3 in Post-COVID-19 Functional Status Scale — PCFS)^17^, who have an education level >9 years and could complete the tests independently. The PCFS was recently translated into Brazilian Portuguese (https://osf.io/tgwe3/) and has been an excellent strategy to assess limitations after SARS-CoV-2 infection. It is graded as follows: 0: no functional limitations, 1: negligible functional limitations, 2: slight functional limitations, 3: moderate functional limitations, 4: severe functional limitation, and D: death. It can be applied in outpatient follow-ups to monitor functional status. The control group comprised individuals who were COVID-19-negative, aged ³18 years old, and were able to understand the tests. The exclusion criteria were individuals with severe and critical COVID-19; a history of mental disorders or current treatment of mental illnesses, such as taking antipsychotics, antidepressants, mood stabilizers, antiepileptics, benzodiazepines, and other drugs that may interfere with the assessment; severe physical illnesses that may interfere with the assessment; history of drug abuse or drug dependence; serious suicidal thoughts; pregnant or lactating women; and individuals with hearing or visual impairments. Participants who did not complete the proposed tests at the time of collection, did not attend reassessments, were exposed to a new COVID-19 infection, or had a neurological or psychiatric disease unrelated to COVID-19 infection during follow-up were excluded from the study.

### Procedures

All tests were performed three times by the research team during 1 year after COVID-19 diagnosis: (a) baseline assessment (after 4 weeks of COVID-19 diagnosis), (b) between 3 and 6 months, and (c) between 6 and 12 months. The individuals reported demographic and clinical variables, such as age, race, and formal education; previous comorbidities were also analyzed, such as hypertension, diabetes, obesity, and sedentary, because preexisting conditions could contribute to slow CRT. Dominance was evaluated using the Edinburgh Handedness Inventory^18^, and cognitive and neuropsychiatric symptoms were evaluated using the MoCA^19^ and Hospital Anxiety and Depression Scale (HADS)^20^, respectively.

### CRT evaluation

**Figure 1:**
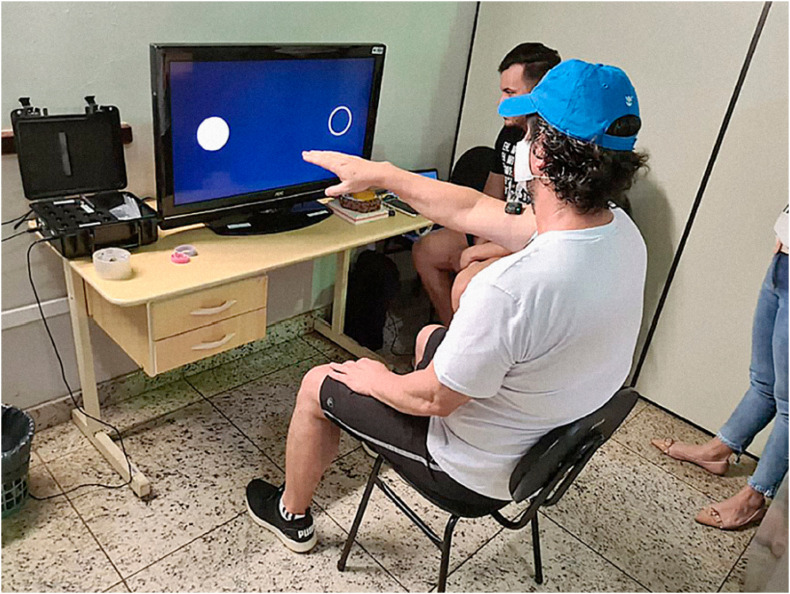
Participant’s position and choice reaction time evaluation.

The CRT was evaluated according to the protocol described by Caires et al.^13^ Participants were seated in a height-adjustable chair in the following positions: hips, knees, and ankles in 90° of flexion; shoulders between 10° and 15° of flexion; elbows in 90° of flexion; and forearms pronated. A smart TV monitor was placed in front of the individual at 100% of the upper limb length. Seat height was adjusted to 100% of the length of the lower limb. Participants had to reach a luminous target projected on a monitor as quickly as possible with their upper limbs and return to the initial position at the end of the stimulus for five trials with the dominant arm (Figure 1).

CRTs were evaluated using electromyographic (EMG) signals according to stimulus onset in the anterior deltoid, brachial biceps, and triceps of the upper limbs. EMG signals were recorded using a Delsys Trigno™ wireless telemetry sensor at 2,000Hz according to the SENIAM protocol (surface EMG for noninvasive assessment of muscles)^21^. The EMG electrode sites were shaved and cleaned with alcohol. EMG onset latency was defined as the time when the EMG amplitude exceeded five standard deviations of the mean of a 100 ms baseline value taken before the onset of the stimulus^22^. A photodiode was used to synchronize the EMG signal with the visual stimulus. The upper limb CRT (measured in milliseconds) was calculated by determining the difference between the photodiode signal and the EMG onset latency in the upper limb while reaching the luminous target.

### Statistical analysis

Data normality was assessed using the Shapiro-Wilk test. Continuous variables were described as means and standard deviations, and categorical variables were expressed as percentages. The outcomes were analyzed using an analysis of variance model with fixed effects due absence of confounders. The goodness of fit was evaluated through the normality of ordinary residuals and homoscedasticity using the Levene’s test. Pairwise post-hoc comparisons were performed using the Bonferroni correction. The Spearman’s test was performed to analyze the correlation between the MoCA, HADS, and CRT values. Statistical significance was set at p<0.05. All statistical analyses were performed by using IBM SPSS Statistics for Windows/Macintosh (version 24.0; IBM Corp., Armonk, NY, USA).

## RESULTS

### Characteristics of the participants

A total of 60 participants (COVID-19: 30; control group: 30) were included. The COVID group had a mean age of 40.5 years and 70% of the individuals were female. The control group had a mean age of 37.9 years and 73.3% of the individuals were female. Among the individuals with COVID-19 evaluated in this study, only five were hospitalized in the acute phase; however, none required intubation or mechanical ventilation. Baseline clinical and demographic data are summarized in Table 1.

**Table 1:** Clinical and demographic profile of both groups.

		COVID-19 (n=30)	Control (n=30)	p-value
Age, year, median (IQR)^1^		40.5 (25-69)	37.9 (21-55)	0.81
Sex^2^, n (%)	Males	9 (30.0)	8 (26.7)	>0.99
	Females	21 (70.0)	22 (73.3)	>0.99
Race^2^, n (%)	White	22 (73.3)	24 (80.0)	0.76
	Black	6 (20.0)	5 (16.7)	>0.99
	Asian	2 (6.7)	1 (3.3)	>0.99
Previous comorbidities, n (%)	Hypertension	13 (43.3)	11 (36.7)	0.79
	Diabetes mellitus	8 (26.7)	6 (20.0)	0.76
	Obesity	5 (16.7)	6 (20.0)	>0.99
	Sedentary	9 (30.0)	10 (33.3)	>0.99
BMI, kg/m^2^, median (IQR)^1^		27.2 (19.0-42.0)	26.4 (20.8-34.8)	0.32
Years of study, median (IQR)^1^		14.3 (12-19)	14.5 (10-22)	0.81
HAD, median (IQR)^1^		10.0 (1.0-19.0)	9.0 (3.0-21.0)	0.37
MoCA, median (IQR)^1^		25.0 (16.0-30.0)	25.0 (21.0-30.0)	0.23
PFCS, median (IQR)^1^		2 (1–3)	0	<0.001

IQR: interquartile range; BMI: body mass index; HAD: Hospital Anxiety and Depression Scale; MoCA: Montreal Cognitive Assessment; PFCS: post-COVID-19 Functional Status Scale.

1 Mann-Whitney U test;

2 χ^2^ test.

In the first evaluation, the individuals presented with the following clinical manifestations: anosmia (18), dysgeusia (15), muscle weakness (21), irritability (10), brain fog (9), headache (8), walking problems (8), arthralgia (7), and myalgia (7). In the second evaluation (3–6 months), the clinical manifestations were hyposmia (16), dysgeusia (13), muscle weakness (12), brain fog (15), and fatigue (16). In the third evaluation (6–12 months), clinical manifestations were hyposmia (12), dysgeusia (8), brain fog (17), and fatigue (16). All individuals (both COVID-19 and control groups) were vaccinated during the follow-up period. Most individuals did not report any adverse effects.

### Outcomes

**Figure 2:**
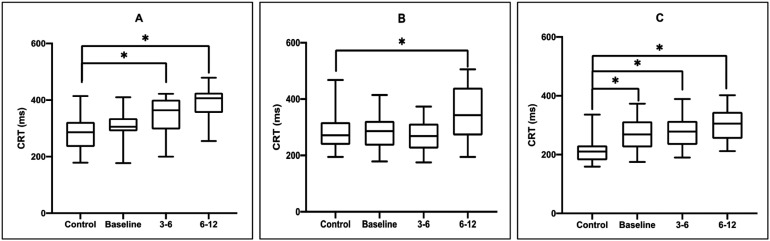
(A) Comparison of the choice reaction time of the anterior deltoid muscle between the control group and the COVID-19 group at baseline, 3-6 months, and 6-12 months after acute infection; (B) Comparison of the choice reaction time of the triceps muscle between the control group and the COVID-19 group at baseline, 3-6 months, and 6-12 months after acute infection; (C) Comparison of the choice reaction time of the brachial biceps muscle between the control group and the COVID-19 group at baseline, 3-6 months, and 6-12 months after acute infection.

The analysis of CRT between the two groups is shown in Figure 2. There was a significant interaction between GROUP and TIME in the CRT of the anterior deltoid [F(2.211, 64.12)=20.40; p<0.001]. Post-hoc analyses showed a significant reduction in CRT of the anterior deltoid in the COVID-19 group at 3-6 (MD, -63.04; 95%CI -103.0 to -23.07; p=0.001) and 6-12 months (MD, -105.2; 95%CI -151.4 to -58.96; p<0.0001) compared to the control group (Figure 2A).

There was a significant interaction between GROUP and TIME in the CRT of the triceps [F(1.979, 57.40)=17.37; p<0.001]. Post-hoc analyses showed significant CRT reduction of triceps in the COVID-19 group at 6-12 months (MD, -67.29; 95%CI -111.8 to -22.82; p=0.002) compared to the control group (Figure 2B).

There was a significant interaction between GROUP and TIME in the CRT of the brachial biceps [F(1.848, 53.59)=42.84; p<0.001). Post-hoc analyses showed a significant CRT reduction of the brachial biceps in the COVID-19 group at 0–3 (MD, -53.16; 95%CI -77.61 to -28.71; p<0.0001), 3-6 (MD, -63.27; 95%CI -88.60 to -37.93; p<0.0001), and 6-12 months (MD, -90.40; 95%CI -117.74 to -63.09; p<0.0001) compared to the control group (Figure 2C).

The mean and standard deviation of the CRT values of the anterior deltoid, triceps, and brachial biceps of all participants are shown in Table 2.

**Table 2:** Mean and standard deviation of choice reaction time values of anterior deltoid, triceps, and brachial biceps of individuals after COVID-19 infection over 1 year and control group.

	COVID-19			Control
	0–3 months	3–6 months	6–12 months	
Anterior deltoid (ms)	307.1±52.30	349.7±61.77	391.9±54.22	286.7±63.11
Triceps (ms)	288.4±61.09	269.3±53.16	353.0±94.61	285.7±62.64
Brachial biceps (ms)	269.3±52.06	279.4±54.79	306.5±57.83	216.1±45.76

Moderate negative correlations were also observed between MoCA and CRT of the anterior deltoid at 6–12 months (r=-0.63; p=0.002) and between MoCA and CRT of the brachial biceps at 6–12 months (r=-0.67; p=0.001). The other variables did not show statistically significant associations.

## DISCUSSION

This study found a reduction in CRT in individuals after COVID-19 infection over 1 year. CRT reduction was found at 3-6 and 6-12 months after acute infection of the anterior deltoid, 6-12 months for triceps, and the brachial biceps in all evaluations compared to the control group. In other words, individuals who have had COVID-19 showed reduced CRT compared to the control group over 1 year. In addition, we observed moderate negative correlations between MoCA and CRT of the anterior deltoid and brachial biceps at 6–12 months.

There are four cognitive processes that can be distinguished in CRT tasks: (1) stimulus perception, (2) stimulus discrimination, (3) response choice, and (4) motor response^23^. RT is important for activities of daily living, requires sensory skills, cognitive processing, and motor performance^24^, and correlates with neuropsychological tests of processing speed and higher order cognitive processes in younger and older adults^25^. Prolonged CRT is associated with decreased cognitive function^23^. Some studies showed that COVID-19 could also alter the brain’s functional connectivity pattern, causing cognitive dysfunction for months after infection resolution^26,27^. Hugon et al. also showed marked attentional and executive cognitive impairment in a patient with mild COVID-19^20^.

Based on the CRT changes observed, can SARS-CoV-2 cause neurological damage to decrease cognitive decision-making in the first year after COVID-19? Can CRT be a resource to diagnose early alterations or post-COVID syndrome? Is CRT a potential predictor of the progression of cognitive loss in long-term COVID-19? The long-term course of these brain lesions and clinical symptoms in mild forms of COVID-19 is difficult to predict. Some authors have mentioned that the evolution toward neurodegenerative diseases could be seen over a prolonged period of time^19,20,28^. In addition, recovery from COVID-19 infection may be associated with particularly pronounced problems in aspects of higher cognitive or “executive” function^29^.

In this study, a correlation was observed between MoCA and CRT only in the period from 6 to 12 months; that is, the lower the MoCA value, the greatest the CRT of the anterior deltoid and brachial biceps muscles. Some authors have presented hypotheses about long-term neurocognitive alterations in individuals who have had COVID-19. These authors reported direct and indirect effects to explain these changes. Regarding direct effects, the authors observed the presence of viral reactivation or hyperactivity of the immune system^30^; and in relation to indirect factors, they report associated extrinsic aspects, such as environmental changes, social isolation, personal and economic factors, as well as lifestyle changes that could later modify neurological and neuropsychiatric function^30^. In addition, associated clinical factors such as fatigue or cardiorespiratory changes can secondarily interfere with cognitive ability; however, these variables were not controlled in this study^31^.

Some limitations of this study should be highlighted. The first is small sample size — which limits the statistical power of our analysis; in order to obtain the best reliability of our analyses, we established strict inclusion criteria to avoid interpretation errors. Even limiting the power of our analysis, the possibility of focusing on a homogeneous subgroup allowed us to minimize the effect of all possible confounders. The second limitation is that due to technical and operational limitations, accuracy and precision were not evaluated during the CRT test, and therefore, they are variables to be controlled in future studies. The third limitation is that objective analysis of fatigue and cardiovascular performance was not performed, which may interfere with cognitive response. Finally, the fourth limitation is that there was no functional MRI analysis to understand the changes at the structural level.

Our findings have important clinical implications in the subacute and chronic phases of COVID-19 because CRT is a simple, low-cost method that can be used as a diagnostic method for brain fog in post-COVID syndrome. Furthermore, these results will help plan and develop multidisciplinary care strategies to improve cognitive performance after COVID-19 infection.

In conclusion, there was a reduction in CRT after acute COVID-19 over 1-year period. CRT reduction was found in the anterior deltoid at 3-6 and 6-12 months, triceps at 6-12 months, and brachial biceps in all evaluations. In addition, a negative correlation was observed between MoCA and CRT only from 6 to 12 months after COVID-19 infection.
